# Human Papillomavirus Vaccination Policies and Discourse on Social Media

**DOI:** 10.1001/jamahealthforum.2025.6425

**Published:** 2026-02-06

**Authors:** Lanyue Zhang, Shouchuang Zhang, Siqi Liu, Weiyan Jian

**Affiliations:** 1 Department of Health Policy and Management, School of Public Health, Peking University, Beijing, China; 2 AI Thrust, Information Hub, The Hong Kong University of Science and Technology (Guangzhou), Guangzhou, China; 3 Key Laboratory of Health System Reform and Governance, National Health Commission of China

## Abstract

**Importance:**

Cervical cancer remains a major public health challenge in China, and human papillomavirus (HPV) vaccine uptake remains far below the global average. Understanding public responses to national policy interventions is critical for promoting equitable vaccine access and uptake.

**Objective:**

To assess the association between 2 national HPV vaccination policies (August 2022 age expansion approval for HPV vaccination [policy 1] and January 2023 National Action Plan for Accelerating the Elimination of Cervical Cancer [policy 2]) in China and public discourse and engagement on social media.

**Design, Setting, and Participants:**

Cross-sectional study using interrupted time-series analysis of publicly available posts by individual users containing HPV-related and vaccine-related keywords from China’s largest text-based social media platform collected between December 2021 and December 2024. Latent Dirichlet allocation topic modeling was used to identify 16 topics grouped into 5 thematic domains.

**Main Outcomes and Measures:**

Changes in the daily trends of public discussions across 5 thematic domains and 16 topics before and after policy implementation using interrupted time-series analysis.

**Results:**

This study analyzed 353 530 HPV-related posts on the social media platform from December 2021 to December 2024. Vaccine accessibility discussions initially increased following the age expansion policy (regression coefficient, 0.08; 95% CI, 0.05-0.12; *P* < .001). After the launch of the National Action Plan, discussions on the theme of vaccine accessibility decreased by 0.11 percentage points per day (95% CI, −0.14 to −0.08; *P* < .001). Conversely, discussions on awareness and knowledge increased by 0.04 percentage points per day (95% CI, 0.03-0.06; *P* < .001), as well as gender and sociocultural factors, which increased by 0.04 percentage points per day (95% CI, 0.02-0.06; *P* < .001).

**Conclusions and Relevance:**

These findings suggest that national HPV vaccination policies in China were associated with shifts in public discourse, including alleviated access concerns and increased health awareness and gender equity discussions. Social media data can provide timely insights into public responses to health interventions and inform strategies to promote equitable HPV vaccine uptake and cervical cancer elimination.

## Key Points

**Question:**

Were the 2 national human papillomavirus (HPV) vaccination policies in China associated with changes in public responses and engagement?

**Findings:**

In this cross-sectional study using 3-year interrupted time-series analysis of 353 530 online posts (2021-2024), discussions on vaccine accessibility decreased substantially after policy implementation, while conversations about health awareness and gender equity issues increased.

**Meaning:**

Results of this study suggest that national HPV vaccination policies in China significantly influenced public perceptions and discussions, supporting efforts to achieve equitable vaccine uptake and cervical cancer elimination.

## Teaser

This cross-sectional study explores whether the 2 national human papillomavirus (HPV) vaccination policies were associated with increased public discourse related to the policies on a social media platform in China.

## Introduction

Human papillomavirus (HPV) infection is the most common sexually transmitted infection worldwide and the leading cause of cervical cancer, which remains a substantial global public health challenge.^1,2,3,4^ HPV vaccination, recommended by the World Health Organization, is a highly effective measure to prevent HPV infection and reduce cervical cancer incidence.^5^ To increase HPV vaccine coverage, many countries have introduced various vaccination policies.^6,7,8^ Yet, the success of these policies also depends on how they are perceived and received by the public.^9^ Conventionally, the effectiveness of public health policies is evaluated primarily through metrics such as final coverage rates.^10,11,12^ However, this approach often overlooks a crucial intermediary layer: the dynamic shifts in public discourse, perceptions, and concerns that occur in response to policy implementation.^13,14^ Public reaction actively shapes the social environment in which policies operate, influencing individual vaccine awareness, attitudes, discussions, and ultimately, the policy’s overall effectiveness.^15,16^ Understanding how policies impact public discussions is crucial for ensuring equitable HPV vaccine uptake and advancing progress toward cervical cancer elimination.^17^

In China, understanding public reception is especially crucial. Although HPV vaccines became available in mainland China in 2016, vaccination coverage remains low.^18,19^ The national HPV vaccination program has a narrow scope, with eligibility restrictions by age, sex and gender, and geographic location, alongside disparities in public perceptions of the vaccine. By 2022, only 10.2% of Chinese females aged 9 to 45 years had received at least 1 dose of the HPV vaccine, far below the global average of 20%.^20^ Recognizing these challenges, China has introduced a series of policy interventions to accelerate vaccine coverage. The 9-valent HPV vaccine was initially approved only for females aged 16 to 26 years, with a strictly limited age eligibility. In August 2022, the National Medical Products Administration expanded the approved age range to 9 to 45 years, substantially relaxing the previous restriction.^19,21^ Subsequently, in January 2023, the National Health Commission launched the Action Plan for Accelerating the Elimination of Cervical Cancer (2023-2030).^22^ This action plan outlined comprehensive national goals to eliminate cervical cancer, emphasizing HPV vaccination coverage, encouragement of HPV vaccination pilot programs, public health education and prevention, and cervical cancer screening and treatment as core strategies. It places special attention on girls, women, and disadvantaged populations (individuals in rural areas or with lower socioeconomic status), and emphasizes the principle of equitable access across urban and rural areas.^23,24^ However, previous studies suggest that public perceptions and responses to national HPV vaccination interventions, particularly concerning accessibility, health equity, and awareness, remain poorly understood.^18,25,26^

Traditionally, surveys and interviews have been used to assess public perceptions of vaccination programs, but these methods often have small sample sizes, recall bias, and reporting delays.^27,28,29^ In contrast, social media platforms offer a current and ongoing source of public discourse, enabling researchers to observe population-level responses to health interventions at scale.^30,31,32^ Previous studies also highlighted that digital public health surveillance through social media listening has emerged as a powerful tool for tracking information dissemination, risk perceptions, and community reactions to health policies.^33,34^ In China, Sina Weibo, the country’s largest microblogging platform with more than 580 million active users, has been widely used for understanding public reactions to policy interventions on influenza and COVID-19.^35,36^ Yet, to our knowledge, few studies have leveraged large-scale social media data to evaluate public responses to national-level HPV vaccination policy implementation.

This study aims to investigate impacts of 2 major HPV vaccination policy interventions in China in terms of public discourse and perceptions as expressed on social media. By analyzing 3 years of online conversations, we seek to capture public attitudes and dynamic responses to these policies; explore barriers related to accessibility, equity, and health awareness; and provide insights for promoting equitable and sustained HPV vaccine uptake.

## Methods

### Study Design

This cross-sectional study used a dataset that included posts related to HPV, cervical cancer, and HPV vaccination, collected from the Sina Big Data System under a data purchase agreement between the School of Public Health, Peking University, and Sina Corporation. Posts were filtered by geotags to ensure relevance to the study area, and only publicly available information shared by users was analyzed. All data were anonymized to exclude sensitive or private information. Keywords for post identification were determined under the guidance of an HPV vaccination expert, with semantic mapping across Chinese and English to ensure comprehensive coverage (eTables 1 and 2 in Supplement 1). Posts published between December 1, 2021, and December 31, 2024, were included. Latent Dirichlet allocation (LDA) topic modeling was applied to process textual data, followed by manual topic classification. Study protocols were approved by the Medical Research Ethics Committee of Peking University Health Science Center. This study was approved by the relevant ethics committee. A waiver of informed consent was granted because the study used anonymized deidentified datasets. The overview of the study design is shown in eFigure 1 in Supplement 1. This study has been reported in line with the Strengthening the Reporting of Observational Studies in Epidemiology (STROBE) consensus criteria.^37^

### Data Collection and Processing

We initially collected 2 906 365 posts from the social media platform using keyword filters combined with temporal and geographic parameters. After excluding 426 015 posts published by nonindividual accounts, 947 818 meaningless information posts (symbols or emojis), 75 446 unknown and overseas posts, 19 013 missing or non-Chinese posts, 44 301 duplicate posts (identical content from the same user), and 471 025 unrelated posts (non-HPV topics), a final dataset of 353 530 posts was retained for analysis. eFigure 2 in Supplement 1 presents the inclusion and exclusion flowchart. Data processing and analysis were conducted using Python version 3.11.7 (Python Software Foundation). Text content was extracted from posts and preprocessed by removing emojis and other nontextual elements. We applied the Jieba Chinese word segmentation library in Python to tokenize each post and removed common stop words with limited analytical value.

### Topic Modeling

An LDA model was implemented in the Python Gensim library to identify latent topics within the Weibo dataset. To mitigate the influence of bot-generated posts and highly similar content, we excluded samples with more than 90% textual similarity (retaining only 1 representative post) and iteratively refined the LDA model. This process continued until artificially clustered topics attributable to bots were no longer detected. To determine the optimal number of topics, we calculated the topic coherence score, which quantifies the semantic consistency of a topic by evaluating the co-occurrence strength of high-frequency words within it. Because higher scores indicate more interpretable and semantically coherent topics, we adopted the topic number with the highest coherence score: *K* = 16 (eFigure 3 in Supplement 1). Finally, each post was assigned to the topic with the highest posterior probability based on all words in the corpus.

### Statistical Analysis

We adopted quantitative descriptive analysis to report the themes and topics of HPV discussions on Weibo. The characteristics of posts were calculated using counts and percentage values. This study applied interrupted time-series (ITS) analysis to evaluate public responses to 2 major national HPV-related policies in China (eMethods in Supplement 1). Additionally, to assess the robustness of our findings, a sensitivity analysis was conducted using the daily total number of HPV-related posts as an alternative outcome in the ITS analysis. Data analyses were conducted in Python version 3.11.7. Two-sided *P* < .05 was considered statistically significant.

## Results

### General Statistics and LDA Topic Modeling

Using LDA, we automatically identified 16 distinct topics from the corpus of posts based on textual content. These topics were then manually grouped into 5 overarching thematic domains: vaccine accessibility, vaccine acceptability, vaccine awareness and knowledge, gender and sociocultural discussions, and pandemic-related narratives. Table 1 shows a summary of each theme and its constituent topics. Overall, females contributed a larger share of posts (270 221 [76.44%]) compared with males (82 558 [23.35%]). Regionally, post volume was highest in eastern (231 681 [65.53%]) and lowest in western (58 499 [16.55%]) provinces. Thematic framework development is described in the eAppendix in Supplement 1, with representative posts and top terms summarized in eTable3 in Supplement 1.

**Table 1.** Themes and Topics of HPV Discussions on the Social Media Platform

Themes and topics	Posts (n = 353 530), No. (%)							
	Policy			Sex^a^		Region		
	Before policy 1	After policy 1 before policy 2	After policy 2	Male	Female	Eastern	Central	Western
Theme 1: vaccine accessibility	87 784 (24.8)	27 752 (7.9)	104 902 (29.7)	42 572 (12.0)	177 343 (50.2)	145 231 (41.1)	38 113 (10.8)	37 048 (10.5)
Vaccine shortage	16 263 (4.6)	2323 (0.7)	21 014 (5.9)	7502 (2.1)	32 002 (9.1)	24 961 (7.1)	7502 (2.1)	7408 (2.1)
Age eligibility restriction	1758 (0.5)	1256 (0.4)	2299 (0.7)	1012 (0.3)	4285 (1.2)	3627 (1.0)	1012 (0.3)	876 (0.3)
Financial burden	3657 (1.0)	1239 (0.4)	4598 (1.3)	2226 (0.6)	2253 (0.6)	6247 (1.8)	2226 (0.6)	1667 (0.5)
Subsidy pilot programs	10 335 (2.9)	2348 (0.7)	5737 (1.6)	4551 (1.3)	13 844 (3.9)	13 107 (3.7)	4551 (1.3)	2642 (0.8)
Insurance coverage	1681 (0.5)	735 (0.2)	4460 (1.3)	1819 (0.5)	5042 (1.4)	4595 (1.3)	1819 (0.5)	1106 (0.3)
Vaccination experience sharing	49 564 (14.0)	19 012 (5.4)	52 877 (15.0)	20 150 (5.7)	100 979 (28.6)	80 082 (22.7)	20 150 (5.7)	20 463 (5.8)
Community-led vaccination services	4526 (1.3)	839 (0.2)	13 917 (3.9)	5312 (1.5)	13 938 (3.9)	12 672 (3.6)	5312 (1.5)	2886 (0.8)
Theme 2: vaccine acceptability	6439 (1.8)	1988 (0.6)	15 448 (4.4)	6830 (1.9)	17 005 (4.8)	15 824 (4.5)	4411 (1.3)	3640 (1.0)
Affirmative vaccine perception	1486 (0.4)	414 (0.1)	6969 (2.0)	2760 (0.8)	6101 (1.7)	6134 (1.7)	2760 (0.8)	1173 (0.3)
Hesitancy and adverse concerns	4953 (1.4)	1574 (0.5)	8479 (2.4)	4070 (1.2)	10 904 (3.1)	9690 (2.7)	4070 (1.2)	2467 (0.7)
Theme 3: awareness and knowledge	5232 (1.5)	1703 (0.5)	32 824 (9.3)	12 983 (3.7)	26 723 (7.6)	26 193 (7.4)	7757 (2.2)	5807 (1.6)
Knowledge of HPV and vaccines	3727 (1.1)	1399 (0.4)	24 942 (7.1)	9849 (2.8)	20 175 (5.7)	19 534 (5.5)	9849 (2.8)	4546 (1.3)
Recognition of vaccine importance	1505 (0.4)	304 (0.1)	7882 (2.2)	3134 (0.9)	6548 (1.9)	6659 (1.9)	3134 (0.9)	1261 (0.4)
Theme 4: gender and sociocultural factors	10 371 (2.9)	2637 (0.8)	40 382 (11.4)	15 928 (4.5)	37 992 (10.8)	34 098 (9.7)	10 992 (3.1)	9263 (2.6)
Women’s health and rights	4416 (1.3)	1031 (0.3)	14 697 (4.2)	6300 (1.8)	13 823 (3.9)	13 124 (3.7)	6300 (1.8)	3121 (0.9)
Male vaccination debate	1493 (0.4)	344 (0.1)	4126 (1.2)	2215 (0.6)	3739 (1.1)	4023 (1.1)	2215 (0.6)	963 (0.3)
Stigma and moral judgment	4462 (1.3)	1262 (0.4)	21 559 (6.1)	6783 (2.0)	20 430 (5.8)	16 951 (4.8)	6783 (1.9)	5179 (1.5)
Theme 5: pandemic-related narratives	5418 (1.5)	2729 (0.8)	7921 (2.2)	4875 (1.4)	11 158 (3.2)	10 335 (2.9)	2992 (0.9)	2741 (0.8)
Vaccination disruption	3174 (0.9)	1715 (0.5)	1476 (0.4)	1313 (0.4)	5040 (1.4)	4081 (1.2)	1313 (0.4)	1132 (0.3)
Pandemic-induced vaccine distrust	2244 (0.6)	1014 (0.3)	6445 (1.8)	3562 (1.0)	6118 (1.7)	6254 (1.8)	3562 (1.0)	1609 (0.5)
Total^b^	115 244 (32.6)	36 809 (10.4)	201 477 (57.0)	82 558 (23.4)	270 221 (76.4)	231 681 (65.5)	63 350 (17.9)	58 499 (16.6)

Abbreviation: HPV, human papillomavirus.

^a^ There are 751 posts that did not report gender; therefore, they are not included in the descriptive statistical analysis.

^b^ Total here refers to the number of 5 themes added together.

Among all themes, vaccine accessibility emerged as the most prevalent, accounting for 220 438 of the total posts (62.35%). This theme included 7 topics: vaccine shortages, age eligibility restrictions, financial burdens, subsidy pilot programs, health insurance relief, vaccination experience sharing, and community-led vaccination services. The second most prevalent theme was “gender and sociocultural factors” (53 390 [15.10%]), followed by “awareness and knowledge” (39 759 [11.25%]), “vaccine acceptability” (23 875 [6.75%]), and finally, “pandemic-related narratives” (16 068 [4.55%]).

### ITS Analysis Results

We report changes in topic trends following each policy to evaluate their immediate and long-term effects (Table 2). After the implementation of policy 1 (August 2022 age expansion approval for HPV vaccination), overall discussions related to vaccine accessibility showed a decline. However, specific topics such as age eligibility restriction (regression coefficient: 1.71; 95% CI, 1.20-2.23; *P* < .001) and insurance coverage (regression coefficient: 0.67; 95% CI, 0.34-0.99; *P* < .001) showed a significant increase in discussion. Similarly, after policy 2 (January 2023 National Action Plan for Accelerating the Elimination of Cervical Cancer) was announced, discussions around insurance coverage increased (regression coefficient: 0.62; 95% CI, 0.06-1.19; *P* < .05), accompanied by a marked increase in community-led vaccination services (regression coefficient: 1.40; 95% CI, 0.76-2.04; *P* < .001).

**Table 2.** Linear Regression Analysis of the Popularity Time Series of the Themes and Topics^a^

Themes and topics	Level change (policy 1)			Slope change (policy 1)			Level change (policy 2)			Slope change (policy 2)		
	Regression coefficient (95% CI)	*P* value	Newey-West SE	Regression coefficient (95% CI)	*P* value	Newey-West SE	Regression coefficient (95% CI)	*P* value	Newey-West SE	Regression coefficient (95% CI)	*P* value	Newey-West SE
Theme 1: vaccine accessibility	−3.76 (−7.49 to −0.04)	.05	1.900	0.08 (0.05-0.12)	<.001	0.017	−2.39 (−5.59 to 0.81)	.14	1.633	−0.11 (−0.14 to −0.08)	<.001	0.016
Vaccine shortage	−1.07 (−2.45 to 0.31)	.13	0.705	0.02 (0.01-0.03)	.01	0.006	0.57 (−0.67 to 1.82)	.37	0.634	−0.02 (−0.02 to −0.01)	<.001	0.005
Age eligibility restriction	1.71 (1.12-2.23)	<.001	0.264	−0.01 (−0.01 to −0.003)	.003	0.003	−0.80 (−1.22 to −0.39)	<.001	0.214	0.01 (0.000-0.01)	.049	0.002
Financial burden	−0.24 (−0.66 to 0.18)	.26	0.214	0.002 (−0.002 to 0.01)	.34	0.002	0.05 (−0.41 to 0.52)	.82	0.238	−0.01 (−0.01 to 0.000)	.03	0.002
Subsidy pilot programs	0.62 (−1.75 to 2.98)	.61	1.206	−0.02 (−0.04 to 0.01)	.17	0.011	1.21 (−0.23 to 2.65)	.10	0.733	0.02 (0.002-0.04)	.03	0.010
Insurance coverage	0.67 (0.34-0.99)	<.001	0.167	0.01 (0.001-0.01)	.01	0.002	0.62 (0.06-1.19)	.03	0.288	−0.003 (−0.01 to 0.001)	.15	0.002
Vaccination experience sharing	−2.98 (−6.24 to 0.28)	.07	1.663	0.04 (0.004-0.08)	.03	0.019	−0.39 (−4.03 to 3.26)	.84	1.859	−0.08 (−0.12 to −0.05)	<.001	0.018
Community-led vaccination services	−0.50 (−1.13 to 0.13)	.12	0.321	0.002 (−0.004 to 0.01)	.47	0.003	1.40 (0.76-2.04)	<.001	0.326	0.01 (0.003-0.01)	<.001	0.002
Theme 2: vaccine acceptability	−4.41 (−5.99 to −2.84)	<.001	0.804	−0.02 (−0.04 to −0.003)	.02	0.008	0.52 (−1.07 to 2.11)	.52	0.811	0.003 (−0.01 to 0.02)	.73	0.008
Affirmative vaccine perception	−0.39 (−0.63 to −0.15)	<.001	0.122	0.001 (−0.001 to 0.003)	.20	0.001	−0.37 (−0.72 to −0.02)	.04	0.177	0.004 (0.002-0.01)	<.001	0.001
Hesitancy and adverse concerns	−2.45 (−3.36 to −1.53)	<.001	0.468	−0.01 (−0.02 to 0.000)	.04	0.004	0.33 (−0.48 to 1.15)	.42	0.416	−0.01 (−0.02 to −0.002)	.01	0.004
Theme 3: awareness and knowledge	2.52 (1.10-3.93)	<.001	0.722	−0.01 (−0.02 to 0.01)	.33	0.008	0.98 (−0.78 to 2.73)	.28	0.895	0.04 (0.03-0.06)	<.001	0.008
Knowledge of HPV and vaccines	1.29 (0.65-1.93)	<.001	0.326	0.001 (−0.01 to 0.01)	.88	0.004	0.54 (−0.53 to 1.61)	.32	0.545	0.02 (0.01-0.02)	<.001	0.004
Recognition of vaccine importance	−0.10 (−0.31 to 0.12)	.39	0.111	0.003 (0.001-0.01)	.01	0.001	−0.02 (−0.37 to 0.34)	.93	0.178	0.01 (0.004-0.01)	<.001	0.001
Theme 4: gender and sociocultural factors	0.87 (−1.91 to 3.66)	.54	1.420	−0.02 (−0.05 to −0.001)	.04	0.012	1.18 (−1.00 to 3.35)	.29	1.111	0.04 (0.02-0.06)	<.001	0.011
Women’s health and rights	0.43 (−0.40 to 1.26)	.31	0.423	−0.01 (−0.011 to 0.000)	.07	0.003	−0.81 (−1.58 to −0.027)	.04	0.397	0.02 (0.01-0.03)	<.001	0.003
Male vaccination debate	0.15 (−0.17 to 0.46)	.36	0.160	−0.003 (−0.01 to 0.000)	.06	0.001	0.82 (0.49-1.14)	<.001	0.167	0.004 (0.002-0.01)	<.001	0.001
Stigma and moral judgment	−0.23 (−1.84 to 1.38)	.78	0.821	−0.02 (−0.03 to −0.01)	.002	0.006	2.87 (1.78-3.96)	<.001	0.556	0.01 (0.003-0.03)	.01	0.006
Theme 5: pandemic-related narratives	4.79 (2.72-6.85)	<.001	1.054	−0.03 (−0.06 to −0.01)	.01	0.012	−0.29 (−2.49 to 1.91)	.80	1.122	0.03 (0.003-0.05)	.03	0.012
Vaccination disruption	2.13 (1.17-3.09)	<.001	0.490	0.01 (−0.002 to 0.023)	.10	0.006	−7.16 (−8.40 to −5.91)	<.001	0.636	−0.02 (−0.03 to −0.003)	.01	0.006
Pandemic-induced vaccine distrust	0.45 (0.13-0.78)	<.006	0.167	−0.004 (−0.01 to 0.000)	.03	0.002	1.14 (0.71-1.58)	<.001	0.222	0.003 (−0.001 to 0.01)	.11	0.002

Abbreviation: HPV, human papillomavirus.

^a^ Regression coefficients, 95% CIs, and Newey-West SEs are measured in %.

However, over time, the trends in public discourse revealed distinct patterns. In terms of gradual effects, there was a significant upward trend in vaccine accessibility discussions following policy 1, with an additional increase of 0.08 percentage points per day (regression coefficient: 0.08; 95% CI, 0.05-0.12; *P* < .001). This growth was driven by heightened conversations about vaccine shortages (regression coefficient: 0.02; 95% CI, 0.01-0.03; *P* < .01), indicating growing demand pressure, and by steady increases in vaccination experience sharing (regression coefficient: 0.04; 95% CI, 0.004-0.08; *P* < .05). Mentions of insurance coverage also showed a moderate yet significant increase (regression coefficient: 0.01; 95% CI, 0.000-0.01; *P* < .05).

**Figure 1.**
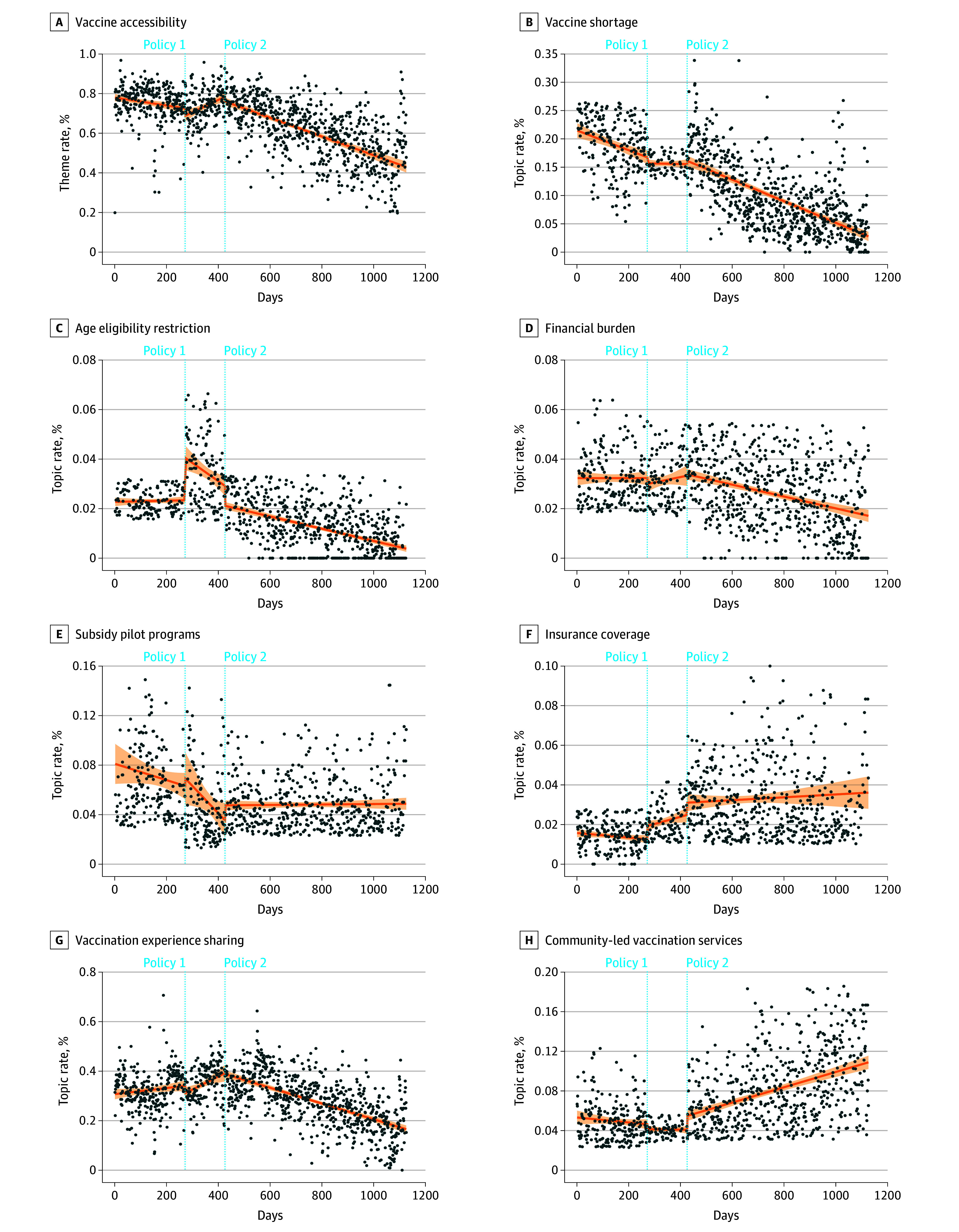
Dot Plots Showing Daily Trends in Public Discussions on Human Papillomavirus Vaccine Accessibility and Its 7 Subtopics on Social Media Posts The shading indicates the 95% CI around the fitted trend line.

In contrast, following policy 2, discussions within the vaccine accessibility theme decreased by 0.11 percentage points per day (regression coefficient: 0.11; 95% CI, −0.14 to −0.08; *P* < .001). Although certain topics such as subsidy pilot programs (regression coefficient: 0.02; 95% CI, 0.002-0.04; *P* < .05) and community-led vaccination services (regression coefficient: 0.01; 95% CI, 0.003-0.01; *P* < .010) continued to increase, discussions of vaccine shortages (regression coefficient: −0.02; 95% CI, −0.03 to −0.01; *P* < .001), financial burden (regression coefficient: −0.01; 95% CI, −0.01 to −0.000; *P* < .05), and vaccination experience sharing (regression coefficient: −0.08; 95% CI, −0.12 to −0.05; *P* < .001) declined significantly over time. The temporal dynamics of discussions within the vaccine accessibility theme and its 7 constituent topics are illustrated in Figure 1.

**Figure 2.**
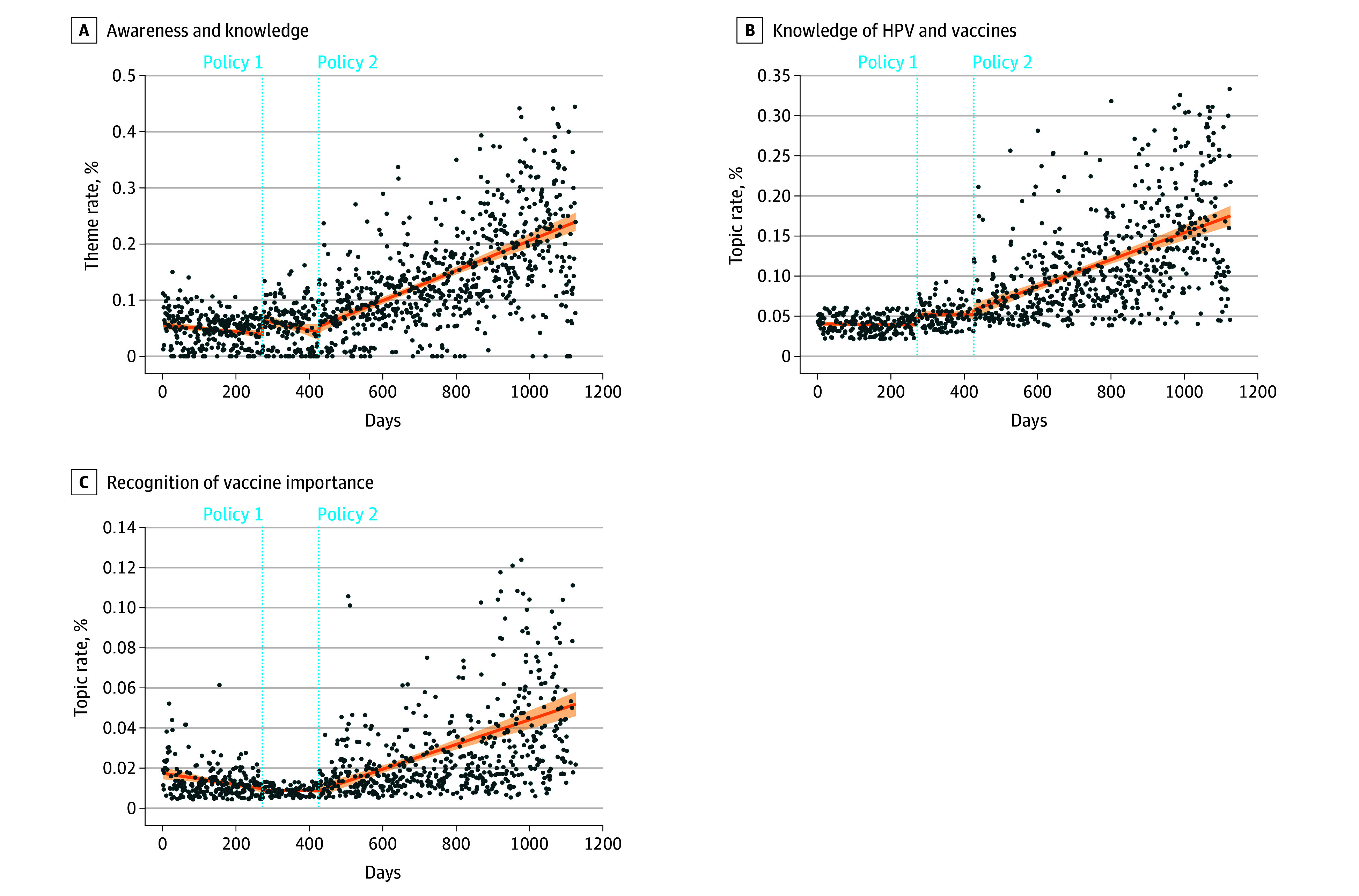
Dot Plots Showing Daily Trends in Public Discussions on Awareness and Knowledge and Its 2 Subtopics on Social Media Posts The shading indicates the 95% CI around the fitted trend line. HPV indicates human papillomavirus.

Within the awareness and knowledge theme, policy 1 was associated with a notable but short-lived increase in public discussions after its implementation, with a significant increase in posts related to HPV knowledge and vaccine value recognition (regression coefficient: 2.52; 95% CI, 1.10-3.93; *P* < .001). However, no significant changes in the subsequent trend were observed following this initial increase. In contrast, policy 2 was associated with a sustained upward trajectory in this theme, as the prevalence of related discussions grew by 0.04 percentage points per day (regression coefficient: 0.04; 95% CI, 0.03-0.06; *P* < .001). Specifically, mentions of knowledge of HPV and vaccines increased steadily at 0.02 percentage points per day (regression coefficient: 0.02; 95% CI, 0.01-0.02; *P* < .001), while recognition of vaccine importance also exhibited a consistent increase of 0.01 percentage points per day (regression coefficient: 0.01; 95% CI, 0.004-0.01; *P* < .001). These temporal trends in discussions around HPV awareness and knowledge, including the 2 constituent topics, are illustrated in Figure 2.

**Figure 3.**
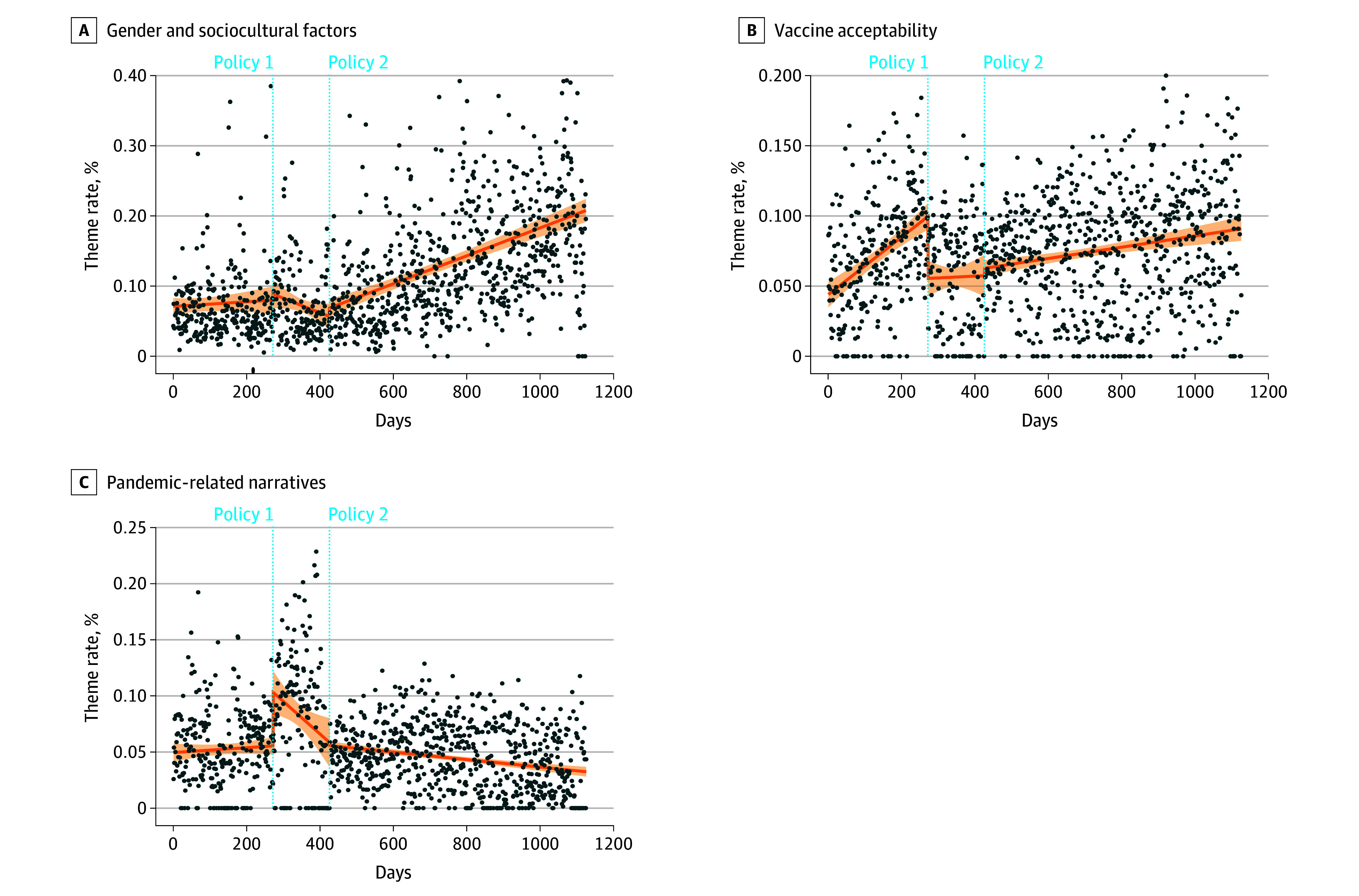
Dot Plots Showing Daily Trends in Public Discussions on Social Media Posts The shading indicates the 95% CI around the fitted trend line.

Both policy 1 and policy 2 demonstrated gradual but contrasting effects with regard to the gender and sociocultural discussions theme. Following policy 1, discussions showed a significant downward trend (regression coefficient: −0.02; 95% CI, −0.05 to −0.000; *P* < .05), while policy 2 was associated with a marked upward trajectory, with discussions increasing by 0.04 percentage points per day (regression coefficient: 0.04; 95% CI, 0.02-0.06; *P* < .001). For the vaccine acceptability theme, only policy 1 showed a statistically significant immediate effect, with discussion prevalence sharply declining by 4.41 percentage points (regression coefficient: −4.41; 95% CI, −5.99 to −2.84; *P* < .001) after its implementation, although no significant long-term changes were observed in the subsequent trend. With regard to the pandemic-related narratives theme, policy 1 showed a pronounced response, with discussions increasing by 4.79 percentage points (regression coefficient: 4.79; 95% CI, 2.72-6.85; *P* < .001) after its announcement, but these declined over time (regression coefficient: −0.03; 95% CI, −0.06 to −0.01; *P* < .010). These temporal trends for gender and sociocultural discussions, vaccine acceptability, and pandemic-related narratives are summarized in Figure 3. eFigures 4-6 in Supplement 1 present further details. Sensitivity analyses yielded results consistent with the primary analyses, with no material changes in the direction or magnitude of the observed associations (eTable 4 and eFigures 7-9 in Supplement 1). Weekly aggregated trends showed similar temporal patterns (eFigures 10-12 in Supplement 1).

## Discussion

HPV infection and cervical cancer prevention remain global public health challenges. Leveraging machine learning techniques, this study analyzed social media discussions around 2 major HPV policies in China to gain insights into public reactions and concerns regarding these health initiatives. Our findings show a sustained decline in discussions related to vaccine accessibility. Although policy 1 temporarily heightened public attention by expanding eligibility and stimulating demand, policy 2, as a more structural and system-oriented policy, likely alleviated public concerns about immediate access barriers, thereby resulting in a steeper downward trend than that observed prior to policy 1. There was a sustained decline in the vaccine accessibility theme, particularly around 3 key topics: vaccine shortages, financial burden, and vaccination experience sharing. The decline in discussions on vaccine shortages and financial burden could suggest that, as policies were introduced and implemented, these key issues have been progressively addressed and became less central in public discourse. The downward trend of vaccination experience sharing may reflect behavioral and psychological shifts that coincided with the normalization of vaccination over time; the sharing of vaccination experiences, once characterized by informational scarcity and a sense of novelty, gradually lost its demonstrative value and social appeal, and contributed to a decline in motivation for sharing such content.^38^

It is important to note that, despite a declining trend over time, discussions around vaccine accessibility consistently remained the most dominant theme in our dataset, echoing findings from previous studies.^39,40^ In China, HPV vaccines have not yet been incorporated into the National Immunization Program.^20^ Although the 2 policy interventions substantially alleviated public concerns about vaccine shortages and costs, which were accompanied by a gradual decrease in the intensity of related discussions, this does not imply that all potential barriers have been eliminated. As of March 2025, HPV vaccine reimbursement policies remain limited to pilot programs in selected provinces.^18^ These programs vary widely in scope and reimbursement levels, which has hindered their ability to fundamentally address the issues of low vaccine uptake and inequitable coverage across the country. Consistent with our findings, public calls for broader and more uniform insurance coverage have gradually intensified over time.

Previous studies have shown that, although early efforts such as free vaccination and school-based programs can substantially boost overall HPV vaccine coverage, uptake among low-income groups and young populations in remote areas often remains well below national averages.^41,42^ This situation also suggests that structural interventions alone, such as improving supply and reducing costs, may improve aggregate coverage but are not necessarily sufficient to narrow these disparities between population subgroups. Thus, China’s 2030 Action Plan for Accelerating the Elimination of Cervical Cancer not only seeks to expand insurance coverage and optimize reimbursement standards but also emphasizes multilevel strategies, including health education and community mobilization, to stimulate proactive vaccination uptake. Such integrated approaches are essential to ensure that HPV vaccination policies translate into equitable and sustainable public health outcomes. Our results further highlight that discussions related to HPV awareness and knowledge showed notable increases following the policy interventions. Previous social media–based studies have found a marked lack of public discourse around vaccine and disease knowledge. In contrast, our analysis found that, prior to the launch of policy 2, discussions of HPV awareness and knowledge remained persistently low.^40,43^ Following the policy’s introduction, however, we observed a significant increase in related discussions, associated with heightened public interest and engagement with HPV vaccine information. The results show that the overall level of public discourse regarding vaccine acceptability remained largely unchanged after the 2 policy interventions, emphasizing the necessity of tailored risk communication efforts to supplement structural policy actions.^44,45,46,47^

In addition, our findings showed a large increase in discussions on gender and sociocultural issues following policy 2, including topics related to women’s health rights, male vaccination debate, stigma, and moral judgment. This pattern highlights that cervical cancer prevention is not merely a biomedical challenge but is deeply intertwined with sociocultural factors. On Chinese social media platforms, HPV is frequently associated with cervical cancer and other female-specific diseases, while the seriousness of HPV infection in males and the importance of male vaccination remain largely overlooked.^40,48^ This has contributed to insufficient risk awareness among males and, in some cases, exposed female vaccine recipients to moralistic judgments and stigmatization in public discourse. The experiences of countries such as Australia, Canada, and Austria demonstrate that, when promotion strategies are overly focused on females, male vaccination coverage tends to lag for prolonged periods, limiting the achievement of herd immunity.^49,50,51,52^ In contrast, incorporating males into national immunization programs and adopting gender-neutral health communication frameworks have been shown to reduce these coverage gaps.^53,54,55^ In our study period (up to the end of 2024), HPV vaccination for males had not yet been approved in mainland China. Nevertheless, we observed persistent public inquiries on social media about “when men will be eligible” and “why male vaccination is necessary,” suggesting that policy 2 not only raised awareness of women’s health but also stimulated broader reflections on vaccine equity and gendered health rights. With the approval of China’s first HPV vaccine for males in January 2025, future efforts should simultaneously strengthen health education targeted at males, emphasizing that HPV prevention is a collective societal responsibility.

### Limitations

This study has several limitations. First, because the data were derived from social media, they may be subject to demographic and geographic biases, with younger and urban users likely overrepresented. Second, social media discussions reflect online discourse rather than actual vaccination behaviors; increased discussion does not necessarily mean higher uptake. Third, findings are based on Weibo data, which may not represent all population segments or other online platforms. Finally, topic misclassification may have occurred due to the ambiguity and diversity of language use, particularly in metaphorical or implicit expressions. Future studies could integrate vaccination coverage data and advanced natural language models to address these challenges.

## Conclusions

By analyzing 3 years of online conversations, our study highlights how national HPV vaccination policies in China were associated with shifts in public discourse on social media related to reducing concerns about vaccine accessibility while fostering greater health awareness and gender-related discussions. Understanding how policies influence public discussions is crucial for promoting equitable HPV vaccine uptake and advancing cervical cancer elimination. As digital communication continues to evolve with emerging technologies, understanding and analyzing social media discourse will become increasingly important for informing responsive and equitable public health strategies.

## Notes

Supplement 1. **eMethods**. Interrupted Time-Series Regression Analysis **eAppendix.** Topic Modeling and Thematic Framework Development **eTable 1.** Keyword List for Weibo Post Search in English **eTable 2.** Keyword List for Weibo Post Search in Mandarin Chinese **eTable 3.** Themes and topics identified from HPV-related Weibo posts **eTable 4.** Sensitivity analysis of total number of HPV-related posts **eFigure 1.** Overview of Study Design **eFigure 2.** Inclusion and exclusion flowchart **eFigure 3.** Comparison of topic numbers and coherence scores curve **eFigure 4.** Daily trends in public discussions on Vaccine Acceptability and its subtopics on Weibo posts **eFigure 5.** Daily trends in public discussions on Gender and Socioculture Factors and its subtopics on Weibo posts **eFigure 6.** Daily trends in public discussions on Pandemic-related Narratives and its subtopics on Weibo posts **eFigure 7.** Sensitivity analysis of daily trends in Vaccine Accessibility discussions using the total number of Weibo posts **eFigure 8.** Sensitivity analysis of daily trends in Awareness and Knowledge discussions using the total number of Weibo posts **eFigure 9.** Sensitivity analysis of daily trends in Gender and Sociocultural Factors, Vaccine Acceptability and Pandemic-related Narratives discussions using the total number of Weibo posts **eFigure 10.** Weekly trends in public discussions on HPV Vaccine Accessibility and its seven subtopics on Weibo posts **eFigure 11.** Weekly trends in public discussions on HPV Awareness and Knowledge and its two subtopics on Weibo posts **eFigure 12.** Weekly trends in public discussions on (A) Gender and Sociocultural Factors, (B) Vaccine Acceptability, and (C) Pandemic related Narratives on Weibo posts

Supplement 2.
Data Sharing Statement
