# Supplementary material for: Human Papillomavirus Vaccination Policies and Discourse on Social Media
Source: JAMA Health Forum. 2026 Feb 6;7(2):e256425. doi: 10.1001/jamahealthforum.2025.6425 (PMC12881989; doi:10.1001/jamahealthforum.2025.6425)
Supplement: Supplement 1. — eMethods. Interrupted Time-Series Regression Analysis eAppendix. Topic Modeling and Thematic Framework Development eTable 1. Keyword List for Weibo Post Search in English eTable 2. Keyword List for Weibo Post Search in Mandarin Chinese eTable 3. Themes and topics identified from HPV-related Weibo posts eTable 4. Sensitivity analysis of total number of HPV-related posts eFigure 1. Overview of Study Design eFigure 2. Inclusion and exclusion flowchart eFigure 3. Comparison of topic numbers and coherence scores curve eFigure 4. Daily trends in public discussions on Vaccine Acceptability and its subtopics on Weibo posts eFigure 5. Daily trends in public discussions on Gender and Socioculture Factors and its subtopics on Weibo posts eFigure 6. Daily trends in public discussions on Pandemic-related Narratives and its subtopics on Weibo posts eFigure 7. Sensitivity analysis of daily trends in Vaccine Accessibility discussions using the total number of Weibo posts eFigure 8. Sensitivity analysis of daily trends in Awareness and Knowledge discussions using the total number of Weibo posts eFigure 9. Sensitivity analysis of daily trends in Gender and Sociocultural Factors, Vaccine Acceptability and Pandemic-related Narratives discussions using the total number of Weibo posts eFigure 10. Weekly trends in public discussions on HPV Vaccine Accessibility and its seven subtopics on Weibo posts eFigure 11. Weekly trends in public discussions on HPV Awareness and Knowledge and its two subtopics on Weibo posts eFigure 12. Weekly trends in public discussions on (A) Gender and Sociocultural Factors, (B) Vaccine Acceptability, and (C) Pandemic related Narratives on Weibo posts [file jamahealthforum-e256425-s001.pdf]

Supplemental Online Content

Zhang L, Zhang S, Liu S, Jian W. Human papillomavirus vaccination policies and discourse on social media. *JAMA Health Forum*. 2026;7(2):e256425. doi:10.1001/jamahealthforum.2025.6425

eMethods. Interrupted Time-Series Regression Analysis

eAppendix. Topic Modeling and Thematic Framework Development

eTable 1. Keyword List for Weibo Post Search in English

eTable 2. Keyword List for Weibo Post Search in Mandarin Chinese

eTable 3. Themes and topics identified from HPV-related Weibo posts

eTable 4. Sensitivity analysis of total number of HPV-related posts

eFigure 1. Overview of Study Design

eFigure 2. Inclusion and exclusion flowchart

eFigure 3. Comparison of topic numbers and coherence scores curve

eFigure 4. Daily trends in public discussions on Vaccine Acceptability and its subtopics on Weibo posts

eFigure 5. Daily trends in public discussions on Gender and Socioculture Factors and its subtopics on Weibo posts

eFigure 6. Daily trends in public discussions on Pandemic-related Narratives and its subtopics on Weibo posts

eFigure 7. Sensitivity analysis of daily trends in Vaccine Accessibility discussions using the total number of Weibo posts

eFigure 8. Sensitivity analysis of daily trends in Awareness and Knowledge discussions using the total number of Weibo posts

eFigure 9. Sensitivity analysis of daily trends in Gender and Sociocultural Factors, Vaccine Acceptability and Pandemic-related Narratives discussions using the total number of Weibo posts

eFigure 10. Weekly trends in public discussions on HPV Vaccine Accessibility and its seven subtopics on Weibo posts

eFigure 11. Weekly trends in public discussions on HPV Awareness and Knowledge and its two subtopics on Weibo posts

eFigure 12. Weekly trends in public discussions on (A) Gender and Sociocultural Factors, (B) Vaccine Acceptability, and (C) Pandemic related Narratives on Weibo posts

This supplemental material has been provided by the authors to give readers additional information about their work.

© 2026 Zhang L et al. *JAMA Health Forum*.

eMethods: Interrupted Time-Series Regression Analysis

We applied Interrupted Time-Series (ITS) to evaluate how two national HPV vaccine policies[1], the Age Expansion Approval (Policy 1) and the Action Plan for Accelerating the Elimination of Cervical Cancer (Policy 2), influenced the prevalence of specific topics in Weibo discussions. The analysis was conducted using Python's stats models library. The ITS regression model was specified as:

$$Y_t = \beta_0 + \beta_1 T_{t0} + \beta_2 X_{t1} + \beta_3 X_{t1} T_{t1} + \beta_4 X_{t1} X_{t2} + \beta_5 X_{t1} X_{t2} T_{t2} + \varepsilon t$$

Where Y_t represents the proportion (%) of a specific topic in posts on day t; T_{t0} denotes the continuous time variable (days since the start of the observation period); T_{t1} and T_{t2} represent days since Policy 1 and Policy 2, respectively; and X_{t1} and X_{t2} are intervention indicators, coded as 0 before and 1 after each policy.

In this formulation, β_0 represents the baseline prevalence at $T = 0$; and β_1 capture the pre-intervention trend (daily change). β_2 and β_4 estimate the immediate level changes following Policy 1 and Policy 2, respectively, while and β_3 and β_5 represent the corresponding slope changes (gradual effects) after each interventions.

Because Policy 2 was introduced while Policy 1 remained in effect, we modeled Policy 2 as an incremental improvement building upon the ongoing effect of Policy 1. The term ($\beta_2 X_{t1} + \beta_3 X_{t1} T_{t1}$) captures the level and trend shifts following Policy 1 up to the introduction of Policy 2, representing the transitional period between the two policies. Similarly, after the implementation of Policy 2, the influence of Policy 1 remained present in the social media discussions. The term ($\beta_4 X_{t1} X_{t2} + \beta_5 X_{t1} X_{t2} T_{t2}$) represents the combined effect following Policy 2, reflecting the joint influence of both policies during the post-Policy 2 period.

As time series data are often autocorrelated, violating the regression assumption of independent residual (εt), we calculated Newey-West standard errors (heteroscedasticity and autocorrelation consistent errors) to ensure robust estimation of parameter confidence intervals.

Reference:

1. Liu, J., et al., *Understanding Human Papillomavirus Vaccination Hesitancy in Japan Using Social Media: Content Analysis.* J Med Internet Res, 2025. **27**: p. e68881.

© 2026 Zhang L et al. *JAMA Health Forum*.

Appendix A: Topic Modeling and Thematic Framework Development

The Latent Dirichlet Allocation (LDA) model was implemented using Python's Gensim library to identify latent topics within the Weibo dataset. To minimize the influence of bot-generated and duplicated posts, samples with over 90% textual similarity were excluded (retaining only one representative post). We iteratively trained LDA models with topic numbers ranging from 5 to 25 and evaluated model performance using the topic coherence score, which quantifies the semantic consistency of each topic based on the co-occurrence strength of high-frequency words. The coherence score curve exhibited a clear peak at K = 16 (see **eFigure 3**), which was selected as the optimal number of topics for subsequent analysis. Each post was then assigned to the topic with the highest posterior probability.

Using LDA, we automatically identified 16 distinct topics from the corpus of Weibo posts based on textual co-occurrence patterns. After the LDA model generated the topics, two independent coders manually reviewed the top 30 keywords and 100 representative posts for each topic to assign interpretable labels and group them into higher-order thematic domains. The manual categorization followed a predefined coding framework derived from prior literature on public health communication and vaccine discourse, emphasizing five key dimensions: (1) accessibility, (2) vaccine acceptability, (3) awareness and knowledge, (4) gender and sociocultural dimensions, and (5) pandemic-related narratives.

The two coders independently proposed topic names and domain groupings and then compared and reconciled any discrepancies through multiple rounds of discussion until full consensus was reached. The final thematic structure was reviewed by an HPV vaccination expert to confirm content validity and ensure consistency with real-world policy discourse.

© 2026 Zhang L et al. *JAMA Health Forum*.

The World Health Organization's (WHO) 3Cs model seeks to describe that individuals' vaccine decision-making and attitudes are primarily influenced by three factors: Convenience (accessibility of vaccination); Confidence (trust or hesitation of the vaccine, health system, and providers), and Complacency (perceptions of disease severity and the necessity of vaccination)[2].

Subsequently, Betsch et al. (2018) built on this foundation to develop the "5C model", which explicitly defines structural barriers as Constraints and adds two additional psychological dimensions: Collective Responsibility (referring to the willingness to be vaccinated to protect others); and Calculation (representing the careful assessment of the risks and benefits associated with vaccination)[3]. This model provides a more systematic explanation of the psychology underlying individuals' vaccine decision-making.

Building upon this theoretical framework foundation, we reviewed previous literature on vaccine policy and health communication, particularly studies analyzing public discourse on the HPV vaccine via social media, and integrated the cultural semantics and discussion contexts specific to Chinese social media platforms[4, 5, 6, 7, 8, 9 10]. Two researchers independently named and categorized the 16 LDA-generated topics, using the high-weight keywords and representative posts for each topic as primary evidence. A consensus was reached after multiple rounds of discussions to ensure semantic consistency.

Ultimately, we merged semantically similar topics into the following five overarching themes, which align with and expand upon the 3C/5C framework. The final thematic structure was also reviewed by an expert in HPV vaccination to confirm its content validity and alignment with real-world policy discourse.

The five final themes are:

© 2026 Zhang L et al. *JAMA Health Forum*.

1. Vaccine Accessibility: Corresponds to Convenience/Constraints in the 3C/5C models, focusing on practical barriers and enablers such as supply shortages, appointment difficulties, cost, and insurance coverage.
2. Vaccine Acceptability: Aligns with Confidence and partially with Calculation, addressing trust or skepticism in vaccines, the healthcare system, and providers, as well as perceptions of safety and efficacy.
3. Awareness and Knowledge: Relates to the informational basis for Complacency and Calculation, covering the public's understanding of HPV, the disease it causes, and the vaccine's function.
4. Gender and Sociocultural Factors: Connects to the social dimension of Collective Responsibility, encompassing discussions on gender equity, social norms, cultural values, and support systems related to vaccination.
5. Pandemic-Related Narratives: Reflects how the COVID-19 pandemic influenced individuals' Calculation, Confidence, and perception of Constraints regarding vaccination decisions.

eTable3 lists representative posts and top terms for each theme and topic.

References:

2. Hardin, B., et al., *Vaccine decision making among people who inject drugs: Improving on the WHO's 3C model of vaccine hesitancy.* Preventive Medicine Reports, 2023. **35**: p. 102341.
3. Betsch, C., et al., *Beyond confidence: Development of a measure assessing the 5C psychological antecedents of vaccination.* PLOS ONE, 2018. **13**(12): p. e0208601.
4. Jamison, A., et al., *Adapting and Extending a Typology to Identify Vaccine Misinformation on Twitter.* Am J Public Health, 2020. **110**(S3): p. S331-s339.

© 2026 Zhang L et al. *JAMA Health Forum*.

5. Allen, J.D., et al., *Feasibility of a twitter campaign to promote HPV vaccine uptake among racially/ethnically diverse young adult women living in public housing.* BMC Public Health, 2020. **20**(1): p. 830.
6. Lama, Y., et al., *Characterizing Trends in Human Papillomavirus Vaccine Discourse on Reddit (2007-2015): An Observational Study.* JMIR Public Health Surveill, 2019. **5**(1): p. e12480.
7. Rai, S., et al., *Detecting and monitoring concerns against HPV vaccination on social media using large language models.* Scientific Reports, 2024. **14**(1): p. 14362.
8. Kornides, M.L., et al., *Exploring content of misinformation about HPV vaccine on twitter.* J Behav Med, 2023. **46**(1-2): p. 239-252.
9. Boucher, J.-C., et al., *HPV vaccine narratives on Twitter during the COVID-19 pandemic: a social network, thematic, and sentiment analysis.* BMC Public Health, 2023. **23**(1): p. 694.
10. Jiang, S., et al., *Social Media Communication about HPV Vaccine in China: A Study Using Topic Modeling and Survey.* Health Communication, 2023. **38**(5): p. 935-946.

© 2026 Zhang L et al. *JAMA Health Forum*.

Appendix B: Supplementary Tables

eTable 1 and eTable 2. Keyword List for Weibo Post Search in English (eTable 1) and Mandarin Chinese (eTable 2)

The keywords are presented in both English (**eTable 1**) and Chinese (**eTable 2**). Given that the original Weibo posts were in Chinese, our keyword search inherently involved semantic mapping across languages. Explanation of AND and OR Relationships:

OR (Logical "OR"): This operator is used to connect related or synonymous terms. For instance, using OR between "hpv" and "human papillomavirus" ensures that posts containing either term will be included in the search results. Similarly, OR connects cancer-related terms like "cervical cancer," "genital cancers," and "warts," ensuring the search captures discussions related to HPV and its associated diseases.

AND (Logical "AND"): This operator is crucial for refining the search by combining terms from different categories. Specifically, when combining (vaccine) with (cervical cancer OR warts OR genital cancers) using AND, the results will include posts that discuss both HPV vaccination and cancer or disease related topics (e.g., cervical cancer, genital warts). This approach ensures that the retrieved posts focus on HPV vaccines and their role in preventing HPV-related cancers.

eTable 1. Keyword List for Weibo Post Search in English

Keyword Group	Keywords
HPV-related terms	human papilloma virus OR human papillomavirus OR hpv OR hvp OR phv
Cervical Cancer and related cancers	cervical cancer OR warts OR genital cancers OR vaginal cancer OR prostate cancer OR penile cancer OR anal cancer
Head and Neck Cancers	head and neck tumors OR head and neck cancer OR oral cancer OR oropharyngeal cancer OR nasopharyngeal cancer OR laryngeal cancer

© 2026 Zhang L et al. *JAMA Health Forum*.

Vaccines and vaccination terms	vaccine OR vaccination OR vaccination injection
9-valent Vaccine	9-valent OR nine valent
Bivalent, Quadrivalent Vaccines	bivalent OR two-valent OR 2-valent OR quadrivalent OR 4-valent
Combination of HPV, Vaccine, and Related Cancers	(human papilloma virus OR hpv OR hvp OR phv) AND (cervical cancer OR warts OR genital cancers)
Combination of Vaccine and HPV-related Terms	(vaccine OR vaccination OR vaccination injection) AND (hpv OR human papilloma virus OR human papillomavirus)

eTable 2. Keyword List for Weibo Post Search in Mandarin Chinese

关键词组	关键词
与 HPV 相关的术语	人乳头瘤 OR 人乳头状瘤 OR 人类乳头状瘤 OR hpv OR hvp OR phv
宫颈癌和相关癌症	宫颈癌 OR 疣 OR 外生殖器癌 OR 外阴癌 OR 阴道癌 OR 前列腺癌 OR 阴茎癌 OR 肛门癌
头颈部癌症	头颈部肿瘤 OR 头颈部癌 OR 口腔癌 OR 口咽癌 OR 鼻咽癌 OR 下咽癌 OR 喉癌
疫苗和接种术语	疫苗 OR 接种 OR 打
九价疫苗	九价 OR 9 价
二价、四价疫苗	二价 OR 两价 OR 2 价 OR 四价 OR 4 价
HPV 和疫苗相关癌症的组合	(人乳头瘤 OR hpv OR 人乳头状瘤) AND (宫颈癌 OR 疣 OR 外生殖器癌)
疫苗和 HPV 相关术语的组合	(疫苗 OR 接种 OR 打) AND (hpv OR 人乳头瘤 OR 人乳头状瘤 OR 人类乳头状瘤)

© 2026 Zhang L et al. *JAMA Health Forum*.

eTable 3. Themes and topics identified from HPV-related Weibo posts

Themes	Topics	Concise Definition	Top terms contributing to the topic model	Representative Weibo posts[a]
**Vaccine Accessibility**	Vaccine shortage	Discussions describing vaccine scarcity, long booking queues, and regional supply gaps.	Shortage, unable, out of stock, appointment, queue, reservation, waiting list, supply, booking, fully booked	The appointment slots for the HPV vaccine are snapped up in seconds... can't get the vaccine at all.
	Age eligibility restriction	Concerns about strict age limits and anxiety about missing eligibility deadlines.	Overage, age, limit, restriction, rejection, deadline, eligibility, rule, age requirement, missed	Only three days left until I'm over the age limit! What should I do if I can't get the HPV vaccine in time?
	Financial burden	Posts emphasizing high vaccine costs and affordability challenges.	Expensive, costly, price, pay, money, unaffordable, burden, cost, high-priced, poor	The HPV vaccine is so expensive, it hurts my wallet.
	Subsidy pilot program	Mentions of government-funded or city-level subsidy pilots providing free or discounted vaccination.	Free, subsidies, government, welfare, pilot program, free dose, support, city policy, benefit, preferential policy	So lucky to be in a government pilot city, my daughter gets the HPV vaccine for free.
	Insurance coverage	Public discussions on the inclusion of HPV vaccination in health insurance schemes	Insurance, health card payment, medical insurance, reimbursed, deduction, claim, policy inclusion, covered, health reimbursement, healthcare	When will the HPV vaccine costs be covered by insurance?!
	Vaccination experience sharing	Individuals sharing personal experiences of booking and receiving, and completing HPV vaccine doses.	Completed, three-doses, second-dose, first-doses, experience, process, appointment, injection, share, feedback	Sharing the process for quickly getting the HPV vaccine~
	Community-led vaccination services	Posts about community health centers organizing vaccination campaigns, education, or outreach.	Community, community-health center, regular education, family doctor, publicity, campaign, publicity, outreach, promotion, poster	Our community has distributed appointment guides for the HPV vaccine.

© 2026 Zhang L et al. *JAMA Health Forum*.

**Vaccine Acceptability**	Affirmative vaccine perception	Expressions of confidence or trust in vaccine safety, protection, and endorsement of vaccination.	Safe, protection, good, nice, recommendation, effective, trust, trustworthy, reliable, protected	Getting the HPV vaccine of course is about protecting ourselves.
	Hesitancy and adverse concerns	Discussions reflecting vaccine hesitancy, safety worries, or doubts.	Hesitation, side-effects, fever, pain, doubts, fear, anxiety, unsafe, allergy, uncertain	Should I get the HPV vaccine? I've heard the side effects can be pretty bad.
**Awareness and Knowledge**	Knowledge of HPV and vaccines	Informational posts explaining HPV infection, transmission, and cancer-prevention benefits.	Cancer, risk, infection, sexually transmitted, prevention, virus, disease, cause, knowledge, health info	The HPV vaccine is the best way to prevent cervical cancer.
	Recognition of vaccine importance	The awareness of HPV vaccination and its long-term health value.	Worth, know, must, health investment, important, priority, necessity, awareness, meaningful, priority	It took a long time, but getting all three doses of the HPV vaccine was totally worth it.
**Gender and Socioculture Factors**	Women's health and rights	Discussions about women's health, gender equality, and empowerment through vaccination.	Women's rights, equality, girls rights, female health, sisters, sis, love, empowerment, protection, fairness	Getting the HPV vaccine is essential for maintaining female health.
	Male vaccination debate	Discussions debating the necessity, feasibility, and effectiveness of HPV vaccination for men and boys.	Male vaccine, man, boy, brothers, husband, necessity, risk, ill, infection, prevention	Do men really need to get the vaccine?!
	Stigma and moral judgment	Posts expressing or countering moral stigma, prejudice, and misconceptions that associate HPV vaccination with promiscuity or a lack of self-respect.	Slut-shaming, stigma, prejudice, moral pollution, lack of self-respect, rumor, shame, conservative, terrible, shameful	Only women who don't love themselves and no self-respect need to get the HPV vaccine.
**Pandemic-related Narratives**	Vaccination disruption	Experiences of vaccination delays due to lockdowns, travel restrictions, or quarantine.	Lockdown, delay, quarantine, interval, yellow-QR-code, postpone, control, zero, risk, outbreak	If the lockdown continues, my HPV vaccine will be delayed again!
	Pandemic-induced vaccine distrust	Posts reflecting spillover distrust toward the HPV vaccine influenced by negative COVID-19 vaccine experiences, misinformation, or conspiracy narratives.	Toxic, fake, covid vaccine, unsold, useless, lie, harmful, conspiracy, scam, poison	COVID-19 vaccine is useless, so the HPV vaccine is even more pointless.

Note: [a] Original posts are in Chinese.

© 2026 Zhang L et al. *JAMA Health Forum*.

eTable 4. Sensitivity analysis of total number of HPV-related posts

Themes and topics	Level Change (Policy 1)				Slope Change (Policy 1)				Level Change (Policy 2)				Slope Change (Policy 2)			
	Coefficient	95% CI	*p*-value	Newey-West S.E.	Coefficient	95% CI	*p*-value	Newey-West S.E.	Coefficient	95% CI	*p*-value	Newey-West S.E.	Coefficient	95% CI	*p*-value	Newey-West S.E.
**Theme 1: Vaccine Accessibility**	-148.304	[-236.831, -59.78]	0.001	45.167	0.270	[0.086, 0.454]	0.004	0.094	46.806	[-30.383, 123.995]	0.235	39.383	-0.177	[−0.282, −0.072]	0.001	0.054
Vaccine shortage	-1.905	[-8.793, -4.983]	0.588	3.514	0.073	[0.02, 0.127]	0.007	0.027	-15.300	[-19.387, -11.214]	0.000	2.085	-0.021	[−0.039, −0.003]	0.024	0.009
Age eligibility restriction	3.181	[0.999, 5.362]	0.004	1.113	-0.034	[-0.054, -0.015]	0.001	0.010	-1.646	[-3.198, -0.094]	0.038	0.792	0.027	[0.009, 0.046]	0.004	0.010
Financial burden	-1.506	[-6.124, 3.113]	0.523	2.356	0.023	[-0.024, 0.070]	0.334	0.024	-0.265	[-2.125, 1.596]	0.780	0.949	0.014	[-0.005, 0.034]	0.153	0.010
Subsidy pilot program	8.178	[-12.393, 28.749]	0.436	10.496	-0.084	[-0.277, 0.109]	0.396	0.098	11.652	[4.904, 18.401]	0.001	3.443	0.211	[0.057, 0.366]	0.007	0.079
Insurance coverage	0.525	[-0.774, 1.824]	0.428	0.663	-0.006	[-0.018, 0.006]	0.313	0.006	4.879	[1.681, 8.077]	0.003	1.631	0.001	[-0.013, 0.015]	0.894	0.007
Vaccination experience sharing	-30.163	[-49.151, -11.176]	0.002	9.688	0.069	[0.001, 0.137]	0.047	0.035	-15.475	[-53.388, 22.438]	0.424	19.344	-0.311	[-0.487, -0.134]	0.001	0.090
Community-led vaccination services	-11.726	[-16.758, -6.694]	0.000	2.567	0.005	[-0.032, 0.041]	0.806	0.019	5.998	[3.311, 8.685]	0.000	1.371	0.015	[0.002, 0.028]	0.020	0.006
**Theme 2: Vaccine Acceptability**	-16.122	[-26.762, -5.483]	0.003	5.428	-0.199	[-0.284, -0.114]	0.000	0.043	6.692	[-2.100, 15.485]	0.136	4.486	0.131	[0.047, 0.214]	0.002	0.043
Affirmative vaccine perception	-2.925	[-4.269, -1.581]	0.000	0.686	-0.003	[-0.012, 0.007]	0.594	0.005	-4.565	[-9.693, 0.563]	0.081	2.616	0.046	[0.019, 0.072]	0.001	0.013
Hesitancy and adverse concerns	-17.860	[-24.951, -10.768]	0.000	3.618	-0.072	[-0.119, -0.025]	0.003	0.024	1.053	[-1.457, 3.563]	0.411	1.281	0.010	[-0.016, 0.036]	0.456	0.013
**Theme 3: Awareness and Knowledge**	0.315	[-8.625, 9.254]	0.945	4.561	-0.093	[-0.160, -0.027]	0.006	0.034	-3.567	[-19.567, 12.433]	0.662	8.164	0.233	[0.135, 0.332]	0.000	0.050
Knowledge of HPV and vaccines	0.731	[-2.395, 3.857]	0.647	1.595	-0.026	[-0.056, 0.005]	0.098	0.016	1.020	[-5.231, 7.272]	0.749	3.190	0.071	[0.036, 0.106]	0.000	0.018
Recognition of vaccine importance	-3.256	[-5.358, -1.155]	0.002	1.072	0.003	[-0.007, 0.014]	0.528	0.005	-0.330	[-2.733, 2.073]	0.788	1.226	0.025	[0.014, 0.036]	0.000	0.006
**Theme 4: Gender and Socioculture Factors**	-4.187	[-29.666, 21.292]	0.747	13.000	-0.275	[-0.474, -0.076]	0.007	0.102	27.588	[4.681, 50.496]	0.018	11.688	0.346	[0.157, 0.536]	0.000	0.097
Women's health and rights	-0.056	[-11.505, 11.394]	0.992	5.842	-0.084	[-0.179, 0.011]	0.081	0.048	-4.491	[-12.783, 3.801]	0.288	4.231	0.173	[0.085, 0.261]	0.000	0.045
Male vaccination debate	1.412	[-3.525, 6.349]	0.575	2.519	-0.033	[-0.077, 0.010]	0.128	0.022	6.568	[2.836, 10.300]	0.001	1.904	0.044	[0.003, 0.086]	0.037	0.021
Stigma and moral judgment	-5.543	[-18.382, 7.295]	0.397	6.550	-0.157	[-0.239, -0.076]	0.000	0.042	25.511	[8.388, 42.634]	0.003	8.736	0.129	[0.054, 0.204]	0.001	0.038
**Theme 5: Pandemic-related Narratives**	8.447	[3.089, 13.805]	0.002	2.733	-0.189	[-0.351, -0.028]	0.022	0.082	9.510	[-0.032, 19.051]	0.051	4.868	0.162	[0.002, 0.322]	0.047	0.082
Vaccination disruption	1.919	[-1.574, 5.412]	0.282	1.782	0.011	[-0.038, 0.059]	0.662	0.025	-18.630	[-23.553, -13.706]	0.000	2.512	-0.032	[-0.080, 0.015]	0.180	0.024
Pandemic-induced vaccine distrust	-1.862	[-3.062, -0.662]	0.002	0.612	-0.014	[-0.025, -0.002]	0.018	0.006	1.238	[0.159, 2.316]	0.024	0.550	0.004	[-0.006, 0.014]	0.413	0.005

Note. CI, Confidence Interval; S.E., Standard Error.

© 2026 Zhang L et al. *JAMA Health Forum*.

Appendix C: Supplementary Figures

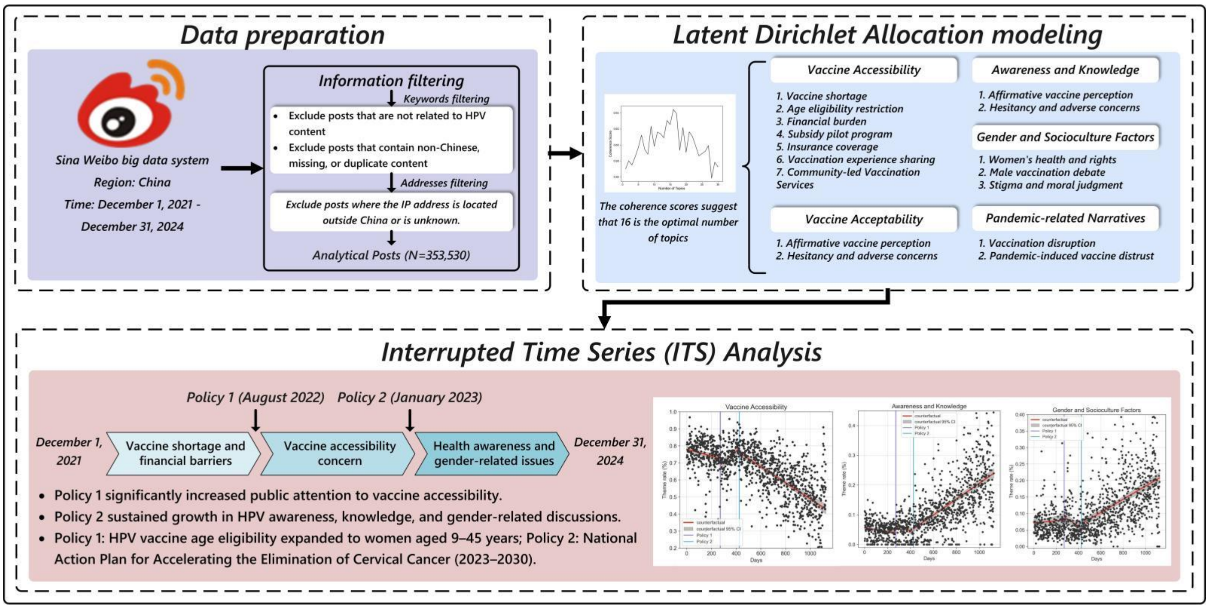

eFigure 1. Overview of Study Design

© 2026 Zhang L et al. *JAMA Health Forum*.

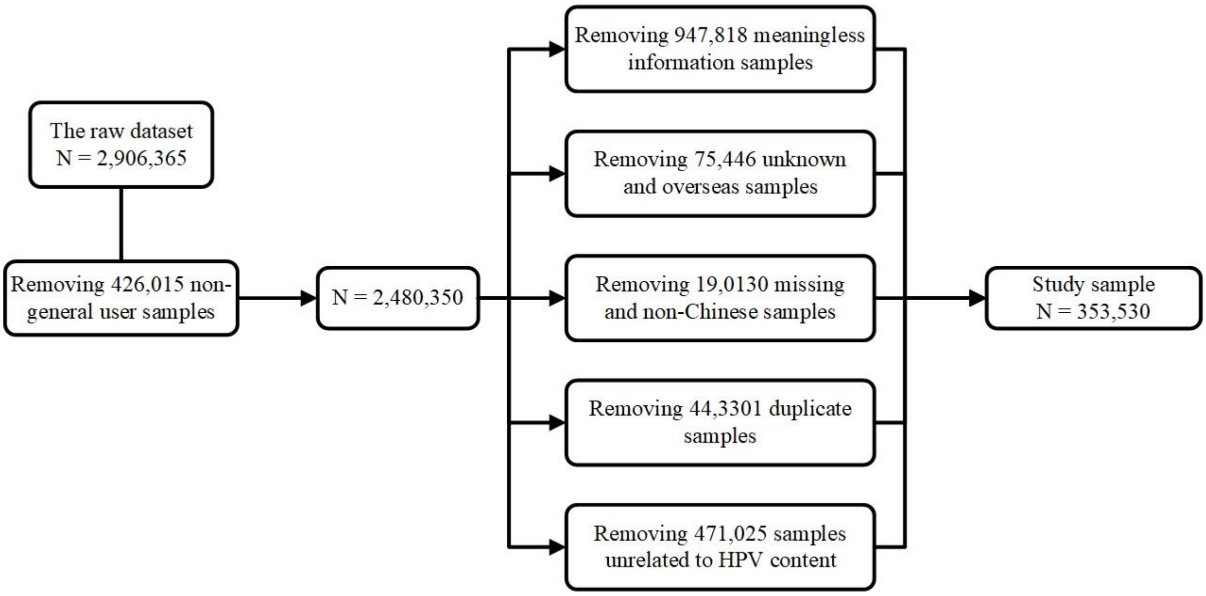

eFigure 2. Inclusion and exclusion flowchart

© 2026 Zhang L et al. *JAMA Health Forum*.

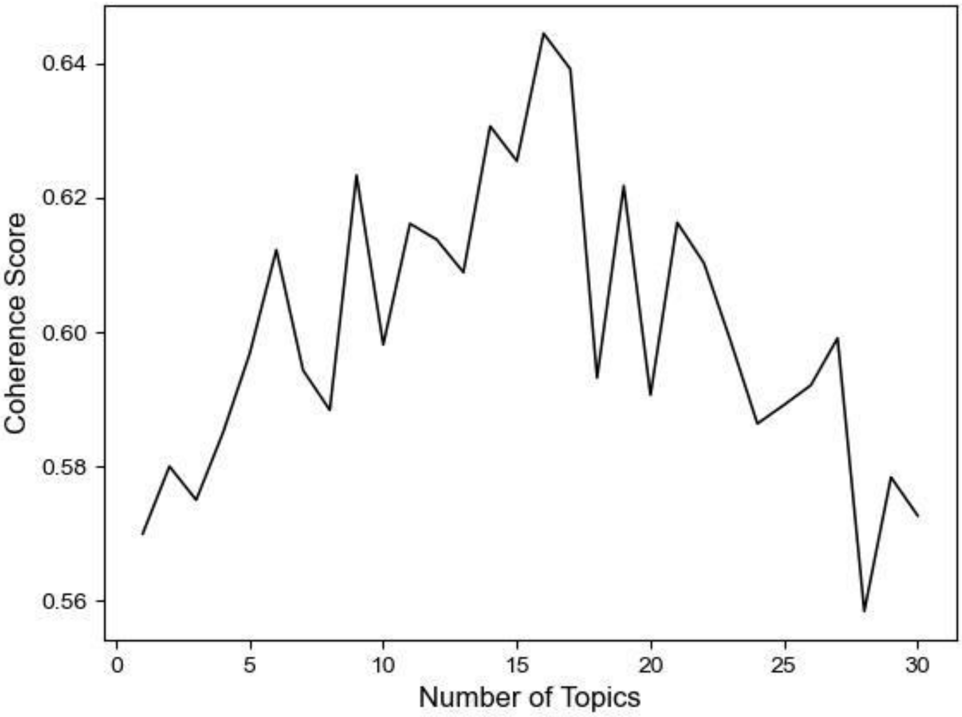

eFigure 3. Comparison of topic numbers and coherence scores curve

© 2026 Zhang L et al. *JAMA Health Forum*.

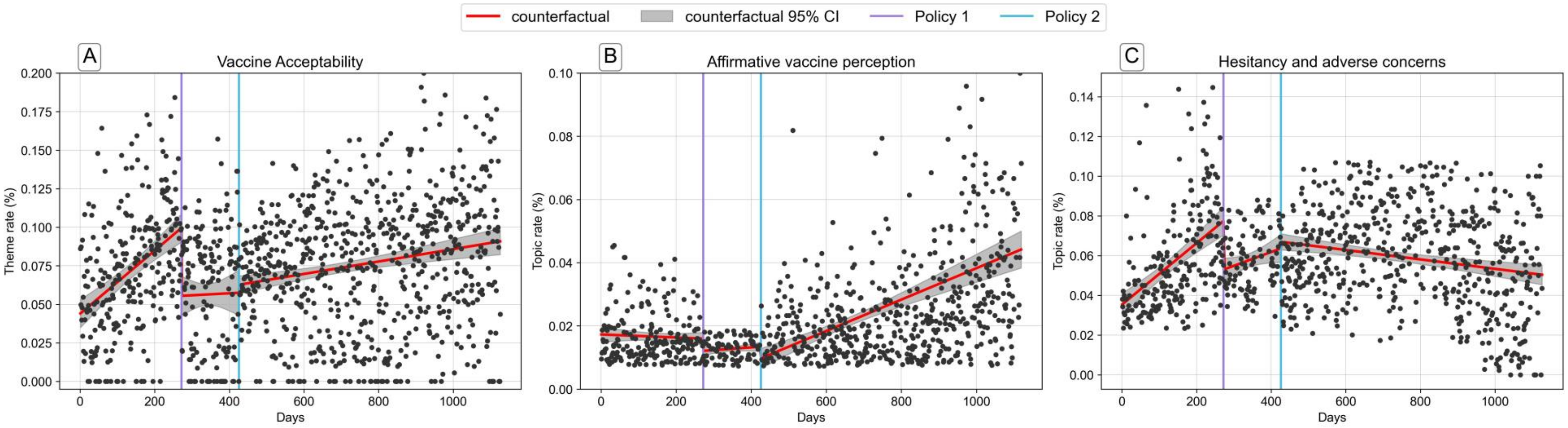

eFigure 4. Daily trends in public discussions on Vaccine Acceptability and its subtopics on Weibo posts

© 2026 Zhang L et al. *JAMA Health Forum*.

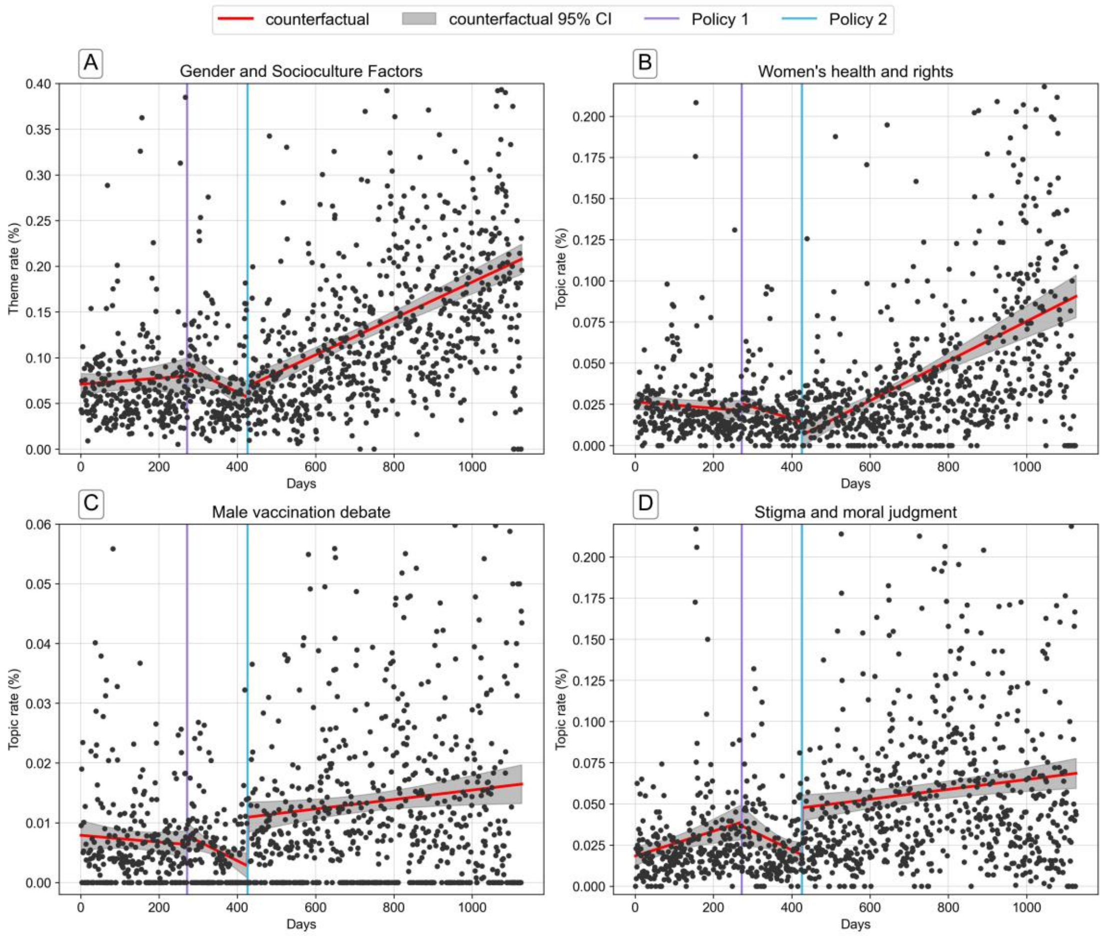

eFigure 5. Daily trends in public discussions on Gender and Socioculture Factors and its subtopics on Weibo posts

© 2026 Zhang L et al. *JAMA Health Forum*.

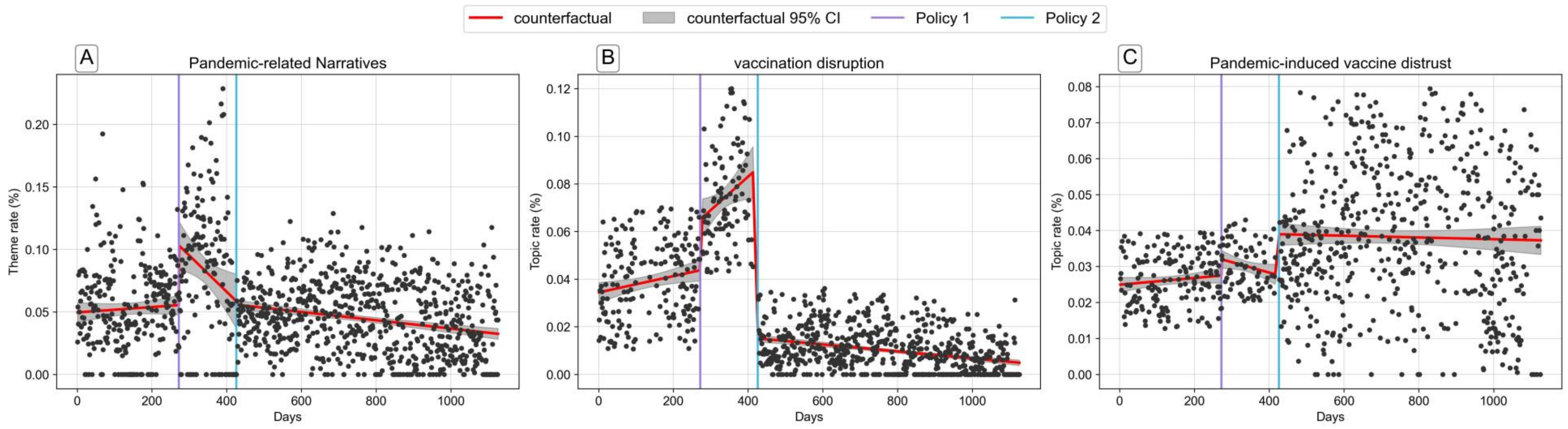

eFigure 6. Daily trends in public discussions on Pandemic-related Narratives and its subtopics on Weibo posts

© 2026 Zhang L et al. *JAMA Health Forum*.

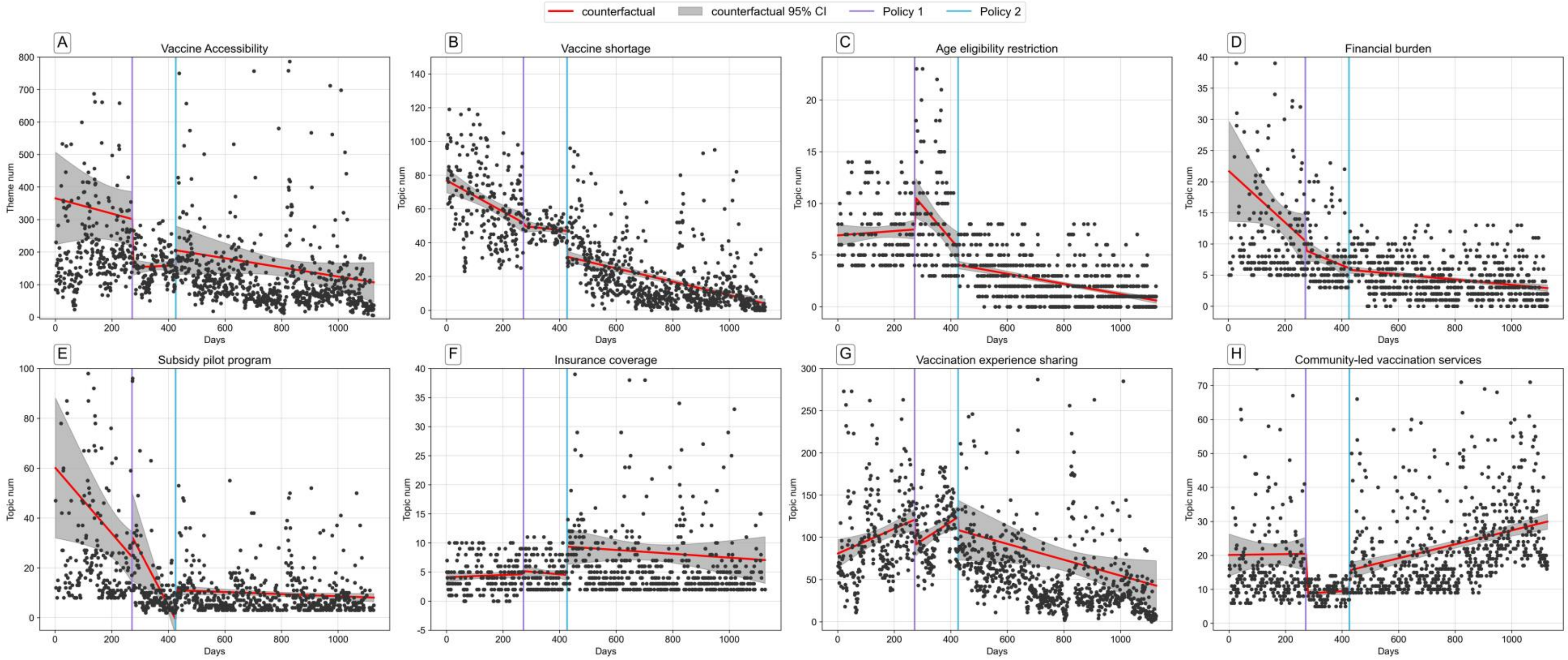

eFigure 7. Sensitivity analysis of daily trends in Vaccine Accessibility discussions using the total number of Weibo posts

© 2026 Zhang L et al. *JAMA Health Forum*.

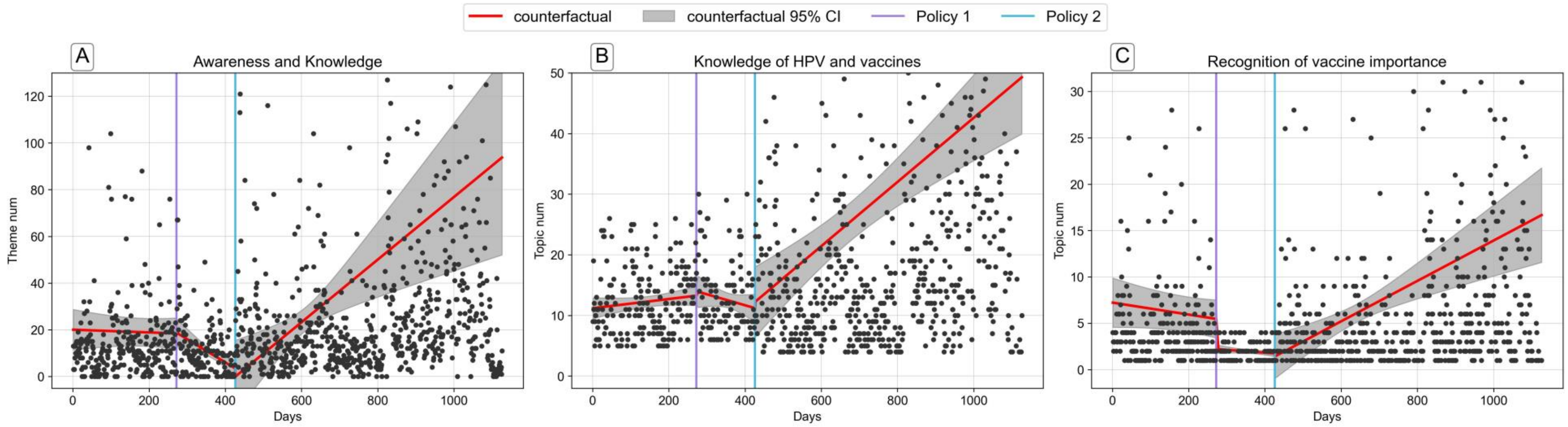

eFigure 8. Sensitivity analysis of daily trends in Awareness and Knowledge discussions using the total number of Weibo posts

© 2026 Zhang L et al. *JAMA Health Forum*.

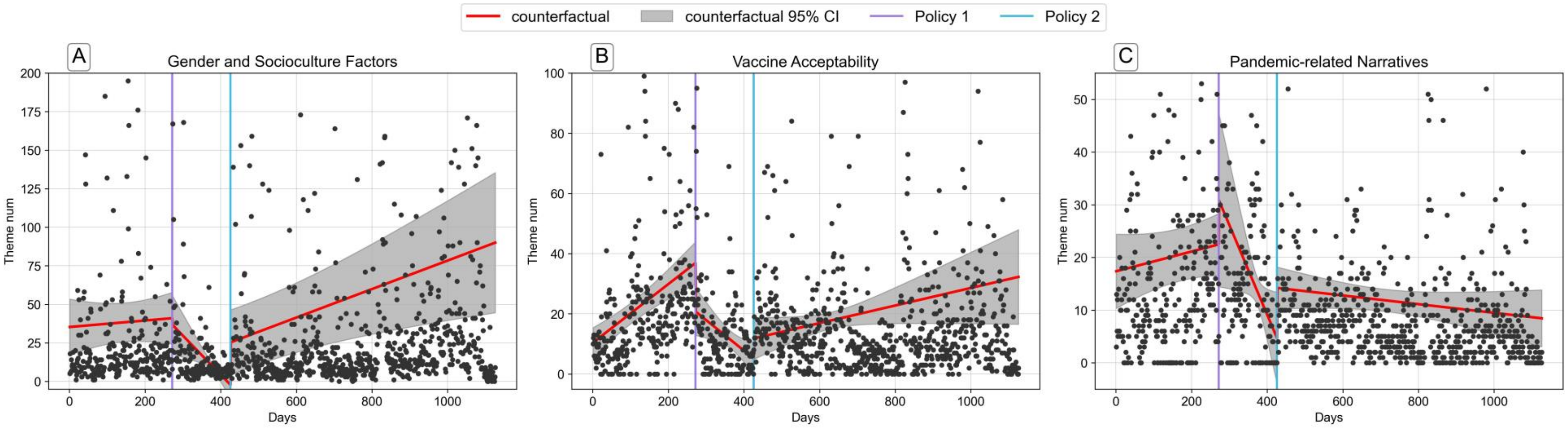

eFigure 9. Sensitivity analysis of daily trends in Gender and Sociocultural Factors, Vaccine Acceptability and Pandemic-related Narratives discussions using the total number of Weibo posts

© 2026 Zhang L et al. *JAMA Health Forum*.

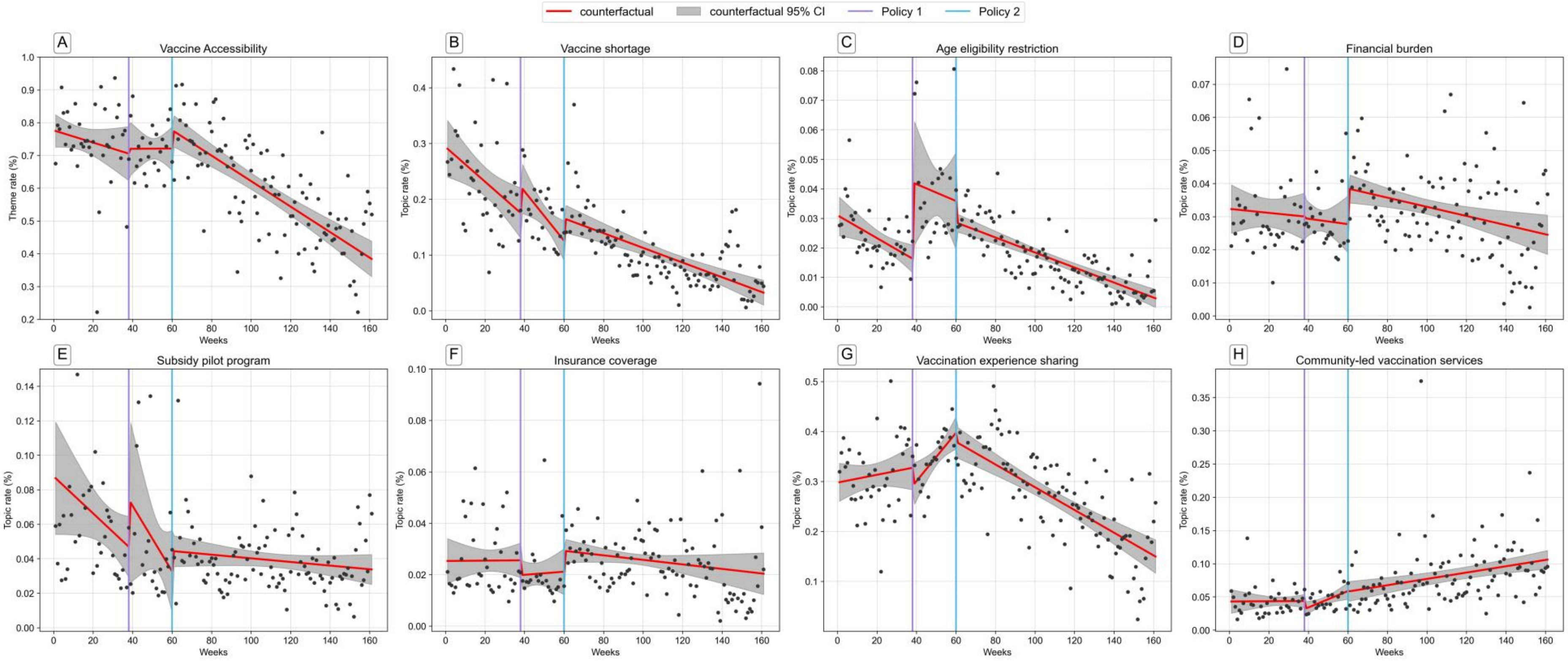

eFigure 10. Weekly trends in public discussions on HPV Vaccine Accessibility and its seven subtopics on Weibo posts

© 2026 Zhang L et al. *JAMA Health Forum*.

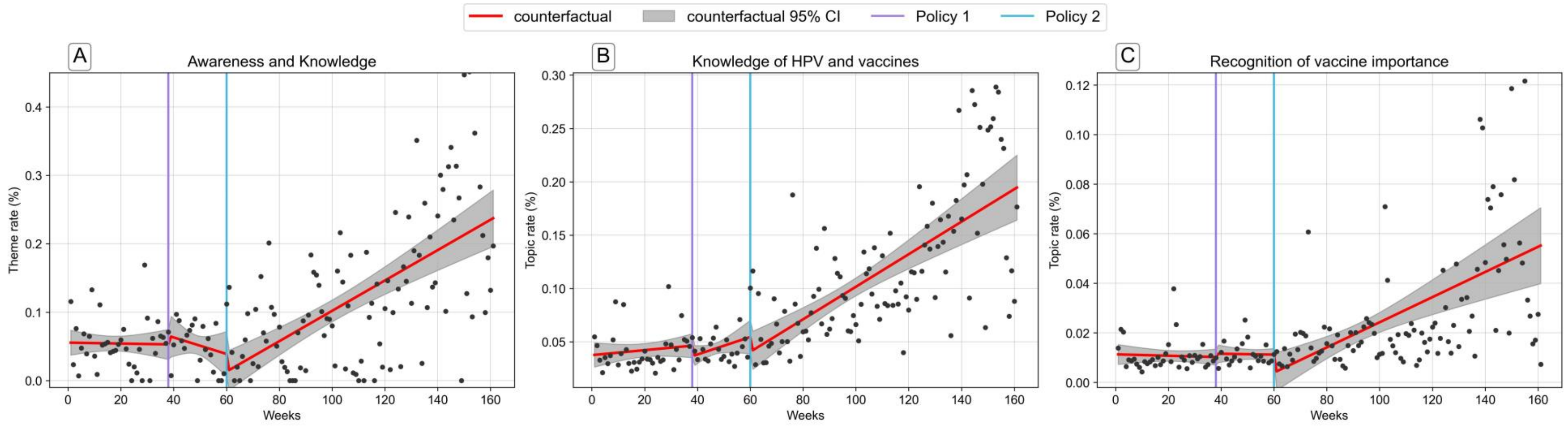

eFigure 11. Weekly trends in public discussions on Awareness and Knowledge and its two subtopics on Weibo posts

© 2026 Zhang L et al. *JAMA Health Forum*.

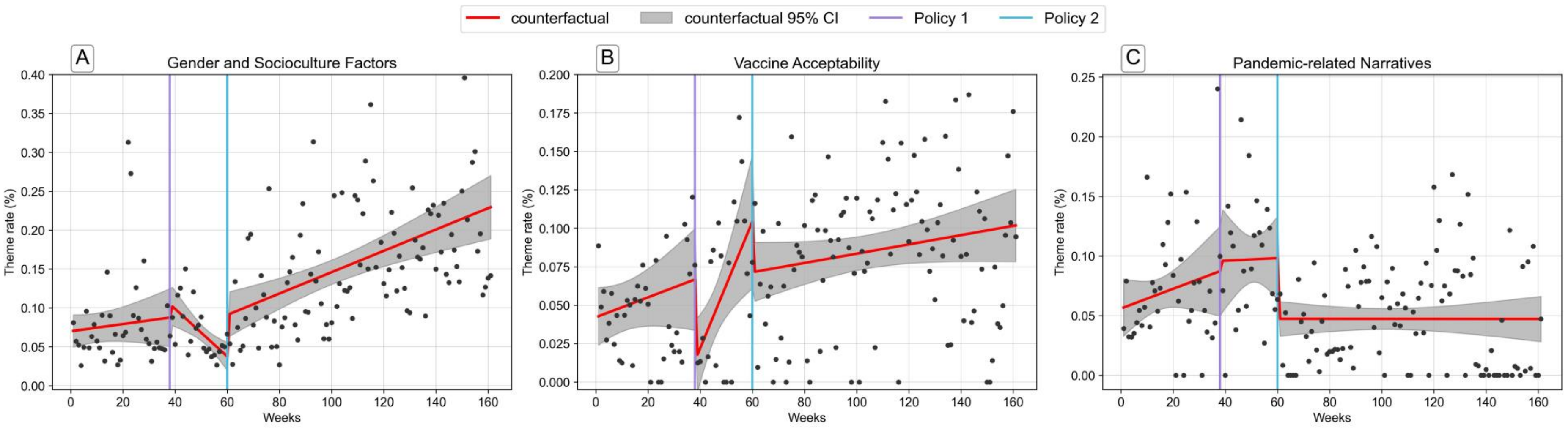

eFigure 12. Weekly trends in public discussions on (A) Gender and Sociocultural Factors, (B) Vaccine Acceptability, and (C) Pandemic-related Narratives on Weibo posts

© 2026 Zhang L et al. *JAMA Health Forum*.
