# Supplementary material for: Human Papillomavirus Vaccination Policies and Discourse on Social Media
Source: JAMA Health Forum. 2026 Feb 6;7(2):e256425. doi: 10.1001/jamahealthforum.2025.6425 (PMC12881989; doi:10.1001/jamahealthforum.2025.6425)
Supplement: Supplement 2. — Data Sharing Statement [file jamahealthforum-e256425-s002.pdf]

Data Sharing Statement

Zhang. Human Papillomavirus Vaccination Policies and Discourse on Social Media. *JAMA Health Forum*. Published February 06, 2026. doi:10.1001/jamahealthforum.2025.6425

Data
Data available: No
